# Bis{μ-2,2′-[(3,4-di­thia­hexane-1,6-di­yl)bis­(nitrilo­methanylyl­idene)]bis­(4-bromo­phenolato)-κ^4^ *O*,*N*,*N*′,*O*′}dicopper(II)

**DOI:** 10.1107/S2056989017017790

**Published:** 2018-01-01

**Authors:** Julia A. Rusanova, Dmytro Bederak, Vladimir N. Kokozay

**Affiliations:** a Department of Chemistry, Taras Shevchenko National University of Kyiv, 64/13 Volodymyrska Street, Kyiv 01601, Ukraine

**Keywords:** crystal structure, dinuclear copper(II) complex, Schiff base, 5-bromo­salicyl­aldehyde, cyste­amine (2-amino­ethanthiol)

## Abstract

The crystal structure of the title compound, [Cu_2_(C_18_H_12_Br_2_N_4_O_2_S_2_)_2_], consists of binuclear complex units which lie across inversion centres and are connected by weak Cu—O coordination bonds forming chains along the *b* axis. The Cu^II^ ion is five-coordinated by two N atoms and two O atoms of the chelating ligand and one symmetry-related O atom forming a square-pyramidal coordination geometry. In the crystal, short S⋯Br contacts connect neighbouring chains into a two-dimensional network parallel to (101).

## Abstract 2

The crystal structure of a novel binuclear copper(II) complex with a dianionic Schiff base derived from 5-bromo­salicylic aldehyde and cyste­amine prepared by direct synthesis is reported.

## Chemical context   

Schiff bases and their metal complexes represent one of the most widely used classes of compound because of their synthetic flexibility and wide range of applications (Mitra *et al.*, 1997 ▸; Bera *et al.*, 1998 ▸; Prabhakaran *et al.*, 2004 ▸). Such complexes having sulfur-containing ligands are of considerable inter­est because of their diverse coordination modes and bridging ability. The formation and cleavage of di­sulfide bonds are known to be important for the biological activity of several sulfur-containing peptides and proteins (Gilbert *et al.*, 1999 ▸; Jacob *et al.*, 2003 ▸).
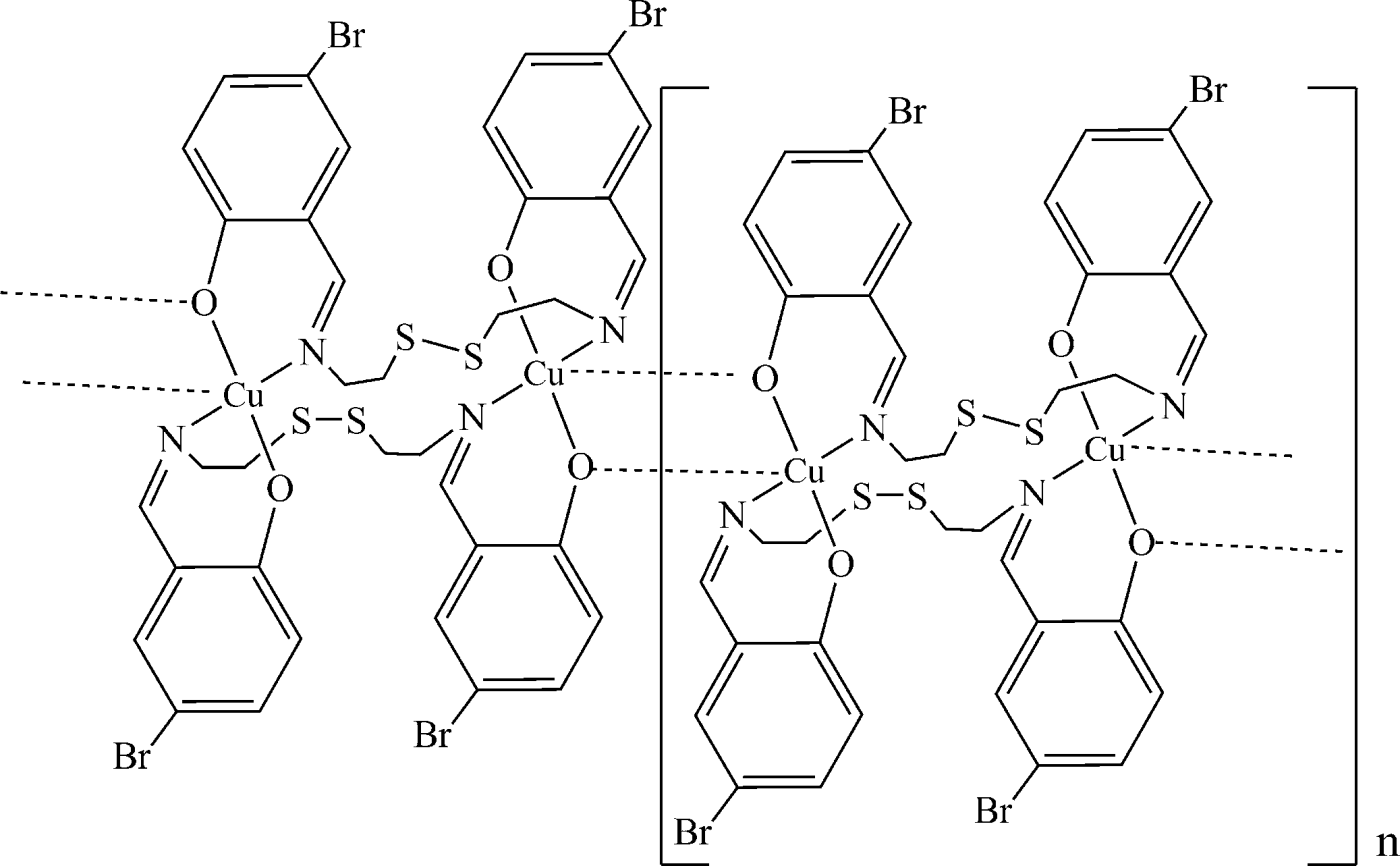


In this study we have continued our investigations in the field of direct synthesis, which is an efficient method to obtain novel polynuclear complexes (Babich & Kokozay, 1997 ▸; Vinogradova *et al.*, 2001 ▸; Nesterova *et al.*, 2008 ▸). The title compound was prepared by direct synthesis starting from zero-valent Cu with a Schiff-base ligand, the product of condensation between 5-bromsalicyl­aldehyde and cyste­amine, formed *in situ* in a methanol/di­methyl­formamide (DMF) mixture.

## Structural commentary   

**Figure 1:**
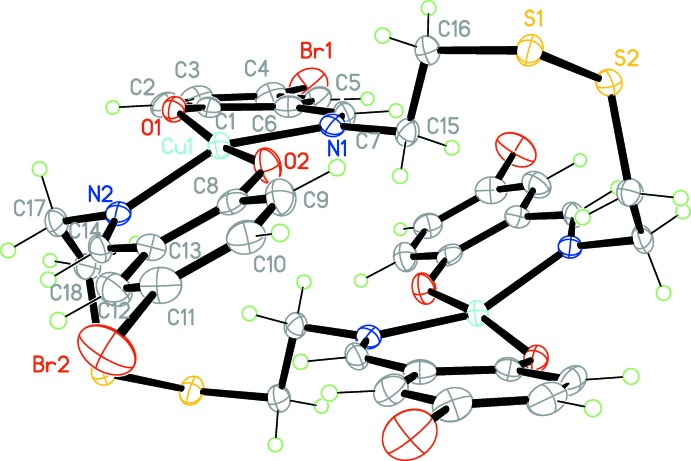
The mol­ecular structure of the binuclear complex unit of the title compound, showing 50% probability displacement ellipsoids. Unlabelled atoms are related to labelled ones by the symmetry operation (2 − *x*, −*y*, 2 − *z*).

**Figure 2:**
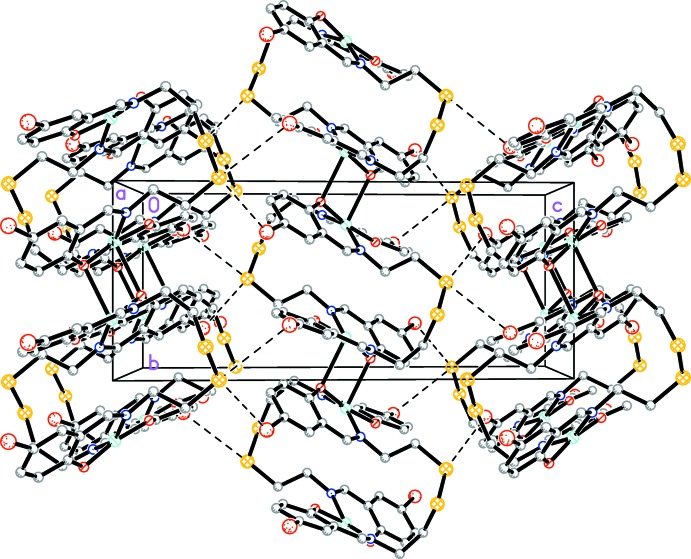
The crystal packing of the title compound viewed along the *a* axis. Short S⋯Br contacts are shown as dashed lines. H atoms are not shown.

In the title compound, binuclear complex units lie across an inversion centre (Fig. 1 ▸). The coordination geometry around the Cu^II^ ion is comparable to that found in copper complexes reported earlier (CSD refcode FEDCIB; Dhar *et al.*, 2005 ▸; Rusanova & Bederak, 2017 ▸). Despite the close structural similarity, neighboring centrosymmetric binuclear fragments are connected by additional weak Cu⋯O (2 − *x*, 1 − *y*, 2 − *z*) coordination bonds with the oxygen atoms of the ligand [2.520 (3) Å] and organized in chains along the *b-*axis direction (Fig. 2 ▸). Thus, each Cu^II^ ion is five-coordinated by two nitro­gen atoms (N1, N2), two oxygen atoms (O1, O2) and one symmetry-related O atom [O1 (2 − *x*, 1 − *y*, 2 − *z*)], forming a distorted square-pyramidal geometry.

The chelating fragments coordinated to the Cu^II^ ions are twisted relative to each other, as defined by the dihedral angle of 28.9 (2)° formed between the mean planes of atoms O1/N1/C1/C6/C7 and O2/N2/C8/C13/C14. The thio­sulfonate moiety is not involved in any metal–ligand inter­actions.

The separation between the two symmetry-related Cu^II^ ions within the binuclear fragment is 5.2161 (11) Å and between neighboring fragments is 3.4458 (11) Å. In general, all bonding parameters and the dimensions of the angles in the title complex are in good agreement with those encountered in related complexes (Dhar *et al.*, 2005 ▸; Zhang *et al.*, 2010 ▸).

## Supra­molecular features   

**Figure 3:**
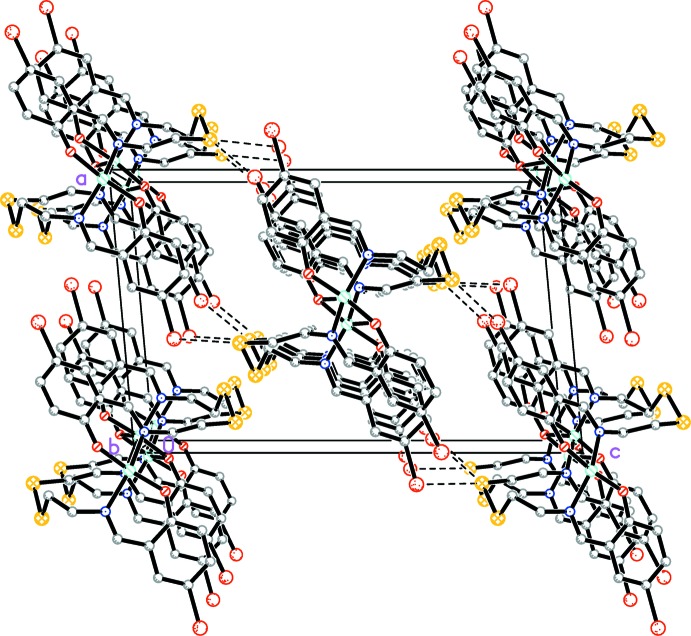
The crystal packing of the title compound viewed along the *b* axis. Short S⋯Br contacts are shown as dashed lines. H atoms are not shown.

In the crystal, short S⋯Br(−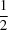 + *x*, 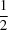 − *y*, 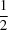 + *z*) contacts with a distance of 3.5108 (13) Å connect neighboring chains, forming a two-dimensional network parallel to (101) (Fig. 3 ▸).

In contrast to the previously reported complex (Rusanova & Bederak, 2017 ▸), there are no hydrogen-bond or π-π stacking inter­actions in the title complex. In terms of C—H⋯Br inter­actions, the inter­molecular C16—H16*B*⋯Br2(*x* + 1, *y*, *z*) distance of 3.03 Å and C17—H17*B*⋯S1(−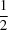 + *x*, 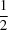 − *y*, −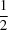 + *z*) distance of 2.95 Å are almost equal to the sum of the van der Waals radii for the atoms involved and may be worthy of note.

## Database survey   

A search of the Cambridge Structural Database (Version 5.38; last update November 2016; Groom *et al.*, 2016 ▸) for related complexes with an amino­ethane­thiol group gave 165 hits, including two closely related structures with a di­sulfide moiety, *viz*. bis­[(μ^2^-sulfato)(6-salicyl­idene­amino-3,4-di­thia­hexyl­ammonium]­copper(II) and bis­(μ^2^-*N*,*N*′-(3,4-di­thia­hexane-1,6-di­yl)bis­(salicylideneiminato)-*N*,*N*′,*O*,*O*′)dicopper(II) (Dhar *et al.*, 2004 ▸, 2005 ▸). The length of the S⋯Br contact in the title compound is in good agreement with those in related complexes (CSD refcodes WEMCAT and QELVIN; Salivon *et al.*, 2006 ▸, 2007 ▸; CSD refcode PODDAO; Xia *et al.*, 2008 ▸)

## Synthesis and crystallization   

A solution of KOH (0.12 g, 2 mmol) in a minimum amount of methanol was added to a solution of amino­ethane­thiol hydro­chloride (0.23g, 2 mmol) in methanol (5 ml) and stirred on an ice bath for 10 min. The white precipitate of solid KCl was removed by filtration and 5-bromsalicyl­aldehyde (0.402 g, 2 mmol) in di­methyl­formamide (10 ml) were added to the filtrate and stirred magnetically for 50 min. Copper powder (0.064 g, 1 mmol) were added to the yellow solution of the Schiff base formed *in situ*, and the resulting deep green–brown solution was stirred magnetically and heated in air at 323–333 K for 2 h, resulting in a dark-green precipitate. Crystals suitable for crystallographic study were grown from a saturated solution in DMF after successive addition of CH_2_Cl_2_. The crystals were filtered off, washed with dry *i*-PrOH and finally dried at room temperature (yield: 20%).

The IR spectrum of the title compound (as KBr pellets) is consistent with the above structural data. In the range 4000–400 cm^−1^ it shows all characteristic functional groups peaks: ν(CH) due to aromatic =C—H stretching at 3000–3100cm^−1^, the aromatic ring vibrations in the 1600–1400 cm^−1^ region, weak S–S absorptions at 500–540 cm^−1^ as well absorbance at 1630 cm^−1^ assigned to the azomethine ν(C=N) group. Analysis calculated for C_36_H_32_Br_4_Cu_2_N_4_O_4_S_4_: C37.28, H 2.78, N 4.83, S 11.06%; found: C 37.32, H 3.01, N 4.70, S 11.10%.

## Refinement   

Crystal data, data collection and structure refinement details are summarized in Table 1 ▸. All hydrogen atoms were placed at calculated positions (C–H = 0.93–0.97 Å) and refined as riding with *U*_iso_(H) = 1.2*U*_eq_(C).

## Supplementary Material

Crystal structure: contains datablock(s) I. DOI: 10.1107/S2056989017017790/lh5862sup1.cif

Structure factors: contains datablock(s) I. DOI: 10.1107/S2056989017017790/lh5862Isup2.hkl

CCDC reference: 1810837

Additional supporting information:  crystallographic information; 3D view; checkCIF report

## Figures and Tables

**Table 1:** Experimental details

Crystal data	
Chemical formula	[Cu_2_(C_18_H_16_Br_2_N_2_O_2_S_2_)_2_]
*M*_r_	1159.61
Crystal system, space group	Monoclinic, *P*2_1_/*n*
Temperature (K)	296
*a*, *b*, *c* (Å)	12.3596 (5), 8.3442 (3), 19.5002 (7)
β (°)	95.156 (2)
*V* (Å^3^)	2002.94 (13)
*Z*	2
Radiation type	Mo *K*α
μ (mm^−1^)	5.31
Crystal size (mm)	0.45 × 0.10 × 0.06

Data collection	
Diffractometer	Bruker SMART APEXII
Absorption correction	Multi-scan *SADABS*
*T*_min_, *T*_max_	0.36, 0.74
No. of measured, independent and observed [*I* > 2σ(*I*)] reflections	18553, 3939, 2644
*R*_int_	0.075
(sin θ/λ)_max_ (Å^−1^)	0.617

Refinement	
*R*[*F* ^2^ > 2σ(*F* ^2^)], *wR*(*F* ^2^), *S*	0.046, 0.098, 1.05
No. of reflections	3939
No. of parameters	244
H-atom treatment	H-atom parameters constrained
Δρ_max_, Δρ_min_ (e Å^−3^)	0.82, −0.66

Computer programs: *SMART* and *SAINT* (Bruker, 2008 ▸), *SHELXT* (Sheldrick, 2015*a* ▸), *SHELXL2016* (Sheldrick, 2015*b* ▸), *SHELXTL* (Sheldrick, 2008 ▸) and *publCIF* (Westrip, 2010 ▸).

## supplementary crystallographic information

### Crystal data

**Table:**

[Cu_2_(C_18_H_16_Br_2_N_2_O_2_S_2_)_2_]	*F*(000) = 1140
*M**_r_* = 1159.61	*D*_x_ = 1.923 Mg m^−^^3^
Monoclinic, *P*2_1_/*n*	Mo *K*α radiation, λ = 0.71073 Å
*a* = 12.3596 (5) Å	Cell parameters from 2534 reflections
*b* = 8.3442 (3) Å	θ = 2.7–23.6°
*c* = 19.5002 (7) Å	µ = 5.31 mm^−^^1^
β = 95.156 (2)°	*T* = 296 K
*V* = 2002.94 (13) Å^3^	Needle, green
*Z* = 2	0.45 × 0.10 × 0.06 mm

### Data collection

**Table:**

Bruker SMART APEXII diffractometer	3939 independent reflections
Radiation source: sealed tube	2644 reflections with *I* > 2σ(*I*)
Graphite monochromator	*R*_int_ = 0.075
phi and ω scans	θ_max_ = 26.0°, θ_min_ = 1.9°
Absorption correction: multi-scan sadabs	*h* = −15→8
*T*_min_ = 0.36, *T*_max_ = 0.74	*k* = −10→10
18553 measured reflections	*l* = −23→24

### Refinement

**Table:**

Refinement on *F*^2^	0 restraints
Least-squares matrix: full	Hydrogen site location: inferred from neighbouring sites
*R*[*F*^2^ > 2σ(*F*^2^)] = 0.046	H-atom parameters constrained
*wR*(*F*^2^) = 0.098	*w* = 1/[σ^2^(*F*_o_^2^) + (0.0426*P*)^2^] where *P* = (*F*_o_^2^ + 2*F*_c_^2^)/3
*S* = 1.05	(Δ/σ)_max_ = 0.004
3939 reflections	Δρ_max_ = 0.82 e Å^−^^3^
244 parameters	Δρ_min_ = −0.66 e Å^−^^3^

### Special details

**Table:**

Geometry. All esds (except the esd in the dihedral angle between two l.s. planes) are estimated using the full covariance matrix. The cell esds are taken into account individually in the estimation of esds in distances, angles and torsion angles; correlations between esds in cell parameters are only used when they are defined by crystal symmetry. An approximate (isotropic) treatment of cell esds is used for estimating esds involving l.s. planes.

### Fractional atomic coordinates and isotropic or equivalent isotropic displacement parameters (Å^2^)

**Table:**

	*x*	*y*	*z*	*U*_iso_*/*U*_eq_	
BR1	1.46222 (5)	0.24549 (7)	0.82836 (3)	0.04443 (19)	
BR2	0.40450 (5)	0.21977 (8)	1.11957 (3)	0.0456 (2)	
CU1	0.95419 (5)	0.30506 (7)	1.00084 (3)	0.01799 (16)	
N1	1.0809 (3)	0.1638 (4)	1.03365 (18)	0.0184 (9)	
N2	0.8169 (3)	0.3643 (4)	0.94366 (18)	0.0174 (9)	
O1	1.0408 (2)	0.4239 (3)	0.94197 (15)	0.0184 (7)	
O2	0.8768 (3)	0.2123 (4)	1.07014 (16)	0.0239 (8)	
S1	1.10722 (11)	−0.01078 (14)	1.23102 (6)	0.0231 (3)	
S2	1.21861 (10)	−0.17845 (15)	1.20755 (6)	0.0229 (3)	
C1	1.1308 (4)	0.3764 (5)	0.9170 (2)	0.0187 (11)	
C2	1.1648 (4)	0.4542 (5)	0.8585 (2)	0.0241 (12)	
H2	1.120805	0.533137	0.836940	0.029*	
C3	1.2610 (4)	0.4166 (6)	0.8324 (2)	0.0279 (13)	
H3	1.282029	0.470919	0.794135	0.033*	
C4	1.3278 (4)	0.2961 (6)	0.8634 (3)	0.0265 (12)	
C5	1.2960 (4)	0.2146 (6)	0.9191 (3)	0.0261 (12)	
H5	1.340017	0.133916	0.939219	0.031*	
C6	1.1973 (4)	0.2517 (5)	0.9464 (2)	0.0205 (11)	
C7	1.1680 (4)	0.1571 (5)	1.0031 (2)	0.0184 (11)	
H7	1.218760	0.081147	1.019849	0.022*	
C8	0.7732 (4)	0.2183 (5)	1.0778 (2)	0.0193 (11)	
C9	0.7380 (4)	0.1630 (6)	1.1407 (2)	0.0260 (12)	
H9	0.788849	0.123649	1.174609	0.031*	
C10	0.6303 (4)	0.1663 (6)	1.1528 (3)	0.0247 (12)	
H10	0.608900	0.131899	1.194901	0.030*	
C11	0.5537 (4)	0.2208 (6)	1.1021 (3)	0.0282 (12)	
C12	0.5833 (4)	0.2746 (6)	1.0406 (3)	0.0287 (13)	
H12	0.530635	0.311494	1.007245	0.034*	
C13	0.6938 (4)	0.2746 (5)	1.0273 (2)	0.0214 (11)	
C14	0.7216 (4)	0.3439 (5)	0.9635 (2)	0.0206 (11)	
H14	0.663784	0.377719	0.933102	0.025*	
C15	1.0733 (4)	0.0487 (5)	1.0908 (2)	0.0191 (11)	
H15A	1.121681	−0.040961	1.085098	0.023*	
H15B	0.999770	0.007538	1.089495	0.023*	
C16	1.1034 (4)	0.1278 (6)	1.1595 (2)	0.0222 (12)	
H16A	1.051164	0.211612	1.166482	0.027*	
H16B	1.174199	0.177672	1.158804	0.027*	
C17	0.8220 (4)	0.4465 (5)	0.8773 (2)	0.0219 (11)	
H17A	0.865362	0.542817	0.884674	0.026*	
H17B	0.749153	0.478879	0.860140	0.026*	
C18	0.8697 (4)	0.3449 (5)	0.8229 (2)	0.0212 (11)	
H18A	0.939036	0.302111	0.841906	0.025*	
H18B	0.883066	0.412405	0.784101	0.025*	

### Atomic displacement parameters (Å^2^)

**Table:**

	*U*^11^	*U*^22^	*U*^33^	*U*^12^	*U*^13^	*U*^23^
BR1	0.0376 (4)	0.0474 (4)	0.0522 (4)	−0.0041 (3)	0.0256 (3)	−0.0010 (3)
BR2	0.0234 (3)	0.0744 (5)	0.0400 (4)	−0.0008 (3)	0.0092 (3)	−0.0080 (3)
CU1	0.0192 (3)	0.0175 (3)	0.0170 (3)	0.0009 (3)	0.0002 (2)	0.0026 (3)
N1	0.028 (2)	0.017 (2)	0.010 (2)	−0.0013 (18)	−0.0003 (18)	−0.0023 (16)
N2	0.024 (2)	0.015 (2)	0.013 (2)	0.0012 (17)	−0.0001 (18)	−0.0034 (16)
O1	0.0209 (19)	0.0170 (18)	0.0174 (17)	0.0007 (14)	0.0019 (15)	0.0013 (14)
O2	0.0197 (19)	0.029 (2)	0.0229 (18)	0.0018 (15)	0.0025 (15)	0.0086 (15)
S1	0.0316 (7)	0.0187 (7)	0.0190 (7)	0.0033 (6)	0.0029 (6)	0.0010 (5)
S2	0.0253 (7)	0.0192 (7)	0.0229 (7)	0.0026 (6)	−0.0044 (6)	−0.0016 (6)
C1	0.022 (3)	0.018 (3)	0.015 (3)	−0.001 (2)	−0.002 (2)	−0.004 (2)
C2	0.037 (3)	0.018 (3)	0.018 (3)	−0.007 (2)	0.004 (2)	0.002 (2)
C3	0.040 (3)	0.026 (3)	0.020 (3)	−0.009 (3)	0.010 (3)	−0.003 (2)
C4	0.029 (3)	0.027 (3)	0.025 (3)	−0.003 (2)	0.010 (2)	−0.006 (2)
C5	0.027 (3)	0.020 (3)	0.031 (3)	−0.001 (2)	0.005 (2)	−0.003 (2)
C6	0.020 (3)	0.022 (3)	0.020 (3)	−0.004 (2)	0.003 (2)	−0.007 (2)
C7	0.017 (3)	0.019 (3)	0.018 (3)	0.004 (2)	−0.002 (2)	0.000 (2)
C8	0.021 (3)	0.014 (3)	0.024 (3)	0.000 (2)	0.004 (2)	−0.006 (2)
C9	0.029 (3)	0.027 (3)	0.022 (3)	0.003 (2)	0.001 (2)	0.004 (2)
C10	0.023 (3)	0.026 (3)	0.026 (3)	−0.006 (2)	0.008 (2)	−0.001 (2)
C11	0.024 (3)	0.033 (3)	0.029 (3)	−0.002 (2)	0.009 (2)	−0.006 (2)
C12	0.021 (3)	0.033 (3)	0.030 (3)	0.001 (2)	−0.003 (2)	−0.003 (2)
C13	0.021 (3)	0.021 (3)	0.022 (3)	−0.002 (2)	−0.002 (2)	−0.002 (2)
C14	0.018 (3)	0.019 (3)	0.023 (3)	0.006 (2)	−0.005 (2)	−0.002 (2)
C15	0.014 (3)	0.017 (3)	0.026 (3)	0.002 (2)	0.002 (2)	0.002 (2)
C16	0.026 (3)	0.021 (3)	0.019 (3)	0.002 (2)	−0.002 (2)	0.003 (2)
C17	0.029 (3)	0.021 (3)	0.015 (3)	0.004 (2)	−0.004 (2)	0.003 (2)
C18	0.024 (3)	0.022 (3)	0.018 (3)	−0.004 (2)	0.001 (2)	0.004 (2)

### Geometric parameters (Å, º)

**Table:**

BR1—C4	1.900 (5)	C6—C7	1.433 (6)
BR2—C11	1.905 (5)	C7—H7	0.9300
CU1—O2	1.890 (3)	C8—C13	1.408 (6)
CU1—O1	1.915 (3)	C8—C9	1.415 (6)
CU1—N2	2.006 (4)	C9—C10	1.372 (6)
CU1—N1	2.018 (4)	C9—H9	0.9300
CU1—O1^i^	2.520 (3)	C10—C11	1.382 (7)
N1—C7	1.277 (5)	C10—H10	0.9300
N1—C15	1.480 (5)	C11—C12	1.362 (7)
N2—C14	1.284 (5)	C12—C13	1.413 (6)
N2—C17	1.471 (5)	C12—H12	0.9300
O1—C1	1.315 (5)	C13—C14	1.441 (6)
O2—C8	1.304 (5)	C14—H14	0.9300
S1—C16	1.808 (5)	C15—C16	1.511 (6)
S1—S2	2.0435 (17)	C15—H15A	0.9700
S2—C18^ii^	1.832 (5)	C15—H15B	0.9700
C1—C2	1.410 (6)	C16—H16A	0.9700
C1—C6	1.415 (6)	C16—H16B	0.9700
C2—C3	1.371 (6)	C17—C18	1.517 (6)
C2—H2	0.9300	C17—H17A	0.9700
C3—C4	1.403 (7)	C17—H17B	0.9700
C3—H3	0.9300	C18—S2^ii^	1.832 (5)
C4—C5	1.369 (7)	C18—H18A	0.9700
C5—C6	1.407 (6)	C18—H18B	0.9700
C5—H5	0.9300		
			
O2—CU1—O1	170.63 (14)	C13—C8—C9	117.8 (4)
O2—CU1—N2	92.36 (15)	C10—C9—C8	121.4 (5)
O1—CU1—N2	91.68 (14)	C10—C9—H9	119.3
O2—CU1—N1	87.85 (14)	C8—C9—H9	119.3
O1—CU1—N1	91.88 (14)	C9—C10—C11	119.7 (5)
N2—CU1—N1	156.01 (14)	C9—C10—H10	120.1
O2—CU1—O1^i^	92.59 (12)	C11—C10—H10	120.1
O1—CU1—O1^i^	78.91 (12)	C12—C11—C10	121.2 (5)
N2—CU1—O1^i^	90.61 (12)	C12—C11—BR2	120.0 (4)
N1—CU1—O1^i^	113.35 (13)	C10—C11—BR2	118.8 (4)
C7—N1—C15	115.8 (4)	C11—C12—C13	120.2 (5)
C7—N1—CU1	122.7 (3)	C11—C12—H12	119.9
C15—N1—CU1	121.2 (3)	C13—C12—H12	119.9
C14—N2—C17	116.0 (4)	C8—C13—C12	119.7 (4)
C14—N2—CU1	123.6 (3)	C8—C13—C14	122.2 (4)
C17—N2—CU1	120.2 (3)	C12—C13—C14	117.9 (4)
C1—O1—CU1	127.1 (3)	N2—C14—C13	127.6 (4)
C8—O2—CU1	129.2 (3)	N2—C14—H14	116.2
C16—S1—S2	103.67 (16)	C13—C14—H14	116.2
C18^ii^—S2—S1	101.41 (16)	N1—C15—C16	110.9 (4)
O1—C1—C2	119.0 (4)	N1—C15—H15A	109.5
O1—C1—C6	123.5 (4)	C16—C15—H15A	109.5
C2—C1—C6	117.5 (4)	N1—C15—H15B	109.5
C3—C2—C1	121.8 (5)	C16—C15—H15B	109.5
C3—C2—H2	119.1	H15A—C15—H15B	108.0
C1—C2—H2	119.1	C15—C16—S1	113.1 (3)
C2—C3—C4	120.0 (4)	C15—C16—H16A	109.0
C2—C3—H3	120.0	S1—C16—H16A	109.0
C4—C3—H3	120.0	C15—C16—H16B	109.0
C5—C4—C3	119.9 (5)	S1—C16—H16B	109.0
C5—C4—BR1	119.9 (4)	H16A—C16—H16B	107.8
C3—C4—BR1	120.2 (4)	N2—C17—C18	113.8 (4)
C4—C5—C6	120.7 (5)	N2—C17—H17A	108.8
C4—C5—H5	119.7	C18—C17—H17A	108.8
C6—C5—H5	119.7	N2—C17—H17B	108.8
C5—C6—C1	120.1 (4)	C18—C17—H17B	108.8
C5—C6—C7	117.3 (4)	H17A—C17—H17B	107.7
C1—C6—C7	122.7 (4)	C17—C18—S2^ii^	113.1 (3)
N1—C7—C6	128.2 (4)	C17—C18—H18A	109.0
N1—C7—H7	115.9	S2^ii^—C18—H18A	109.0
C6—C7—H7	115.9	C17—C18—H18B	109.0
O2—C8—C13	124.2 (4)	S2^ii^—C18—H18B	109.0
O2—C8—C9	118.0 (4)	H18A—C18—H18B	107.8

Symmetry codes: (i) −*x*+2, −*y*+1, −*z*+2; (ii) −*x*+2, −*y*, −*z*+2.
